# Clinical outcomes for *Clostridioides difficile* associated diarrhea in inflammatory bowel disease patients versus non-IBD population: A retrospective cohort study

**DOI:** 10.1097/MD.0000000000032812

**Published:** 2023-02-10

**Authors:** Genady Drozdinsky, Alaa Atamna, Hagar Banai, Haim Ben-Zvi, Jihad Bishara, Noa Eliakim-Raz

**Affiliations:** a Internal Medicine E, Rabin Medical Center Beilinson Campus, Petah-Tikva, Israel; b Sackler Faculty of Medicine, Tel Aviv University, Tel Aviv, Israel; c Infectious Diseases Unit, Rabin Medical Center, Beilinson Hospital, Petah-Tikva, Israel; d Gastroenterology Unit, Rabin Medical Center, Beilinson Hospital, Petah-Tikva, Israel; e Department of Clinical Microbiology, Rabin Medical Center, Beilinson Hospital, Petah-Tikva, Israel.

**Keywords:** antibiotic, *Clostridioides difficile* infection (CDI), inflammatory bowel disease (IBD)

## Abstract

Patients with inflammatory bowel disease (IBD) have a higher incidence of *Clostridioides difficile* infection (CDI). Previous studies have demonstrated negative clinical outcomes in IBD patients with CDI compared to patients without CDI. The clinical presentation of CDI is indistinguishable from IBD exacerbation, thus posing a frequent clinical dilemma on the role of *Clostridioides* infection in the testing, diagnosis, and treatment of these patients. To compare clinical outcomes of CDI in patients with IBD to those without IBD. Retrospective cohort of adult patients admitted to Rabin Medical Center Israel between the years 2014 and 2020 with a concurrent diagnosis of IBD and CDI. Matching 1:2 was performed between the IBD patients and the non-IBD population with respect to age and sex. Sixty-seven patients with IBD and 134 patients without IBD were included in the study. The groups’ median age was 40.6 (interquartile range [IQR] of 29.8–68.9), with 45.8% male and 54.2% female. The non-IBD group had a higher Charlson score with 2 (IQR 0; 5) versus 0 (IQR 0; 4) in the IBD group (*P* value <.01). Patients with IBD had more exposure to systemic antibiotics, 71.1% versus 26.3% (*P* value <.01). In a multivariable analysis we found no difference in 90-day mortality and rate of relapse between the 2 study groups with an odds ratio of 1.709 (95% confidence interval 0.321–9.905) and odds ratio of 0.209 (95% confidence interval 0.055–1.513) respectively. In our cohort patients with IBD who present with diarrhea and concomitant CDI have similar rates of relapse and mortality compared with patients without IBD.

## 1. Introduction

*Clostridioides difficile* (CD, formerly known as *Clostridium difficile*) is a gram-positive, fastidious, and spore-forming bacteria responsible for toxin-mediated infectious colitis associated with antibiotic use and healthcare interaction.^[1]^ *C. difficile* infection (CDI) can manifest in a wide spectrum of severity from mild diarrhea to fatal pseudomembranous colitis and toxic megacolon. CDI is a nosocomial infection that can spread in an endemic and sporadic fashion. In 2019, CDI was diagnosed in 223,900 patients in the USA and accounted for 12,800 deaths.^[2]^

Inflammatory bowel disease (IBD) is a spectrum of chronic, systemic immune-mediated diseases affecting primarily but not only the gastrointestinal tract.^[3]^ The prevalence of IBD is on the rise in recent years. In a systematic review of the literature, Molodecky et al estimated an annual worldwide incidence of 5 to 24.3 per 100,000 person-years.^[4]^ The occurrence of CDI is higher in patients with IBD.^[5]^ In a retrospective study, by Rodemann et al, analyzing patients diagnosed with CDI showed an odds ratio (OR) of 2.9 (confidence interval [CI] 95% 2.1–4.1) for IBD versus non-IBD patients. In his study, the stool CD toxin was diagnosed in the first 48 hours of admission, implying the disease was mainly community-acquired.^[6]^ The association between IBD and CDI is not completely understood and most likely comprise of an interplay between interrupted gastrointestinal mucosal barrier, frequent antibiotic exposure, recurrent hospitalizations, altered nutritional status, genetic predisposition, microbiome, and immune factors.^[7]^ In a study evaluating 6932 patients with IBD in Greece, the prevalence of CDI was statistically significantly higher than in non-IBD hospitalized patients with 30/401 versus 309/6531.^[8]^ Known risk factors for CDI are usually more common in IBD patients notably immunosuppression and systemic antibiotic exposure.

The sometimes indistinguishable clinical presentation of the 2 clinical syndromes,^[9]^ complicates diagnosis, may cause delays in treatment, and presents dilemmas in treatment approaches. The long and short-term clinical outcomes of patients with CDI and IBD are worse than those of IBD exacerbation alone.^[10–12]^ Interestingly, Gupta et al in a study evaluating 92 patients with IBD found that toxin enzyme-linked immunosorbent assay (ELISA) positive patients had a higher rate of response to anti-*Clostridioides* antibiotics compared to ELISA test negative and nucleic acid amplification test (NAAT) positive patients and had lower chances of requiring IBD therapy escalation.^[13]^ In the post hoc analysis of MODIFY I/II, patients with a positive ELISA test had a more severe disease on one hand and a higher chance of treatment response on the other hand.^[14]^ Similar results were demonstrated by Avni et al in the non-IBD population with lower 30-day all-cause mortality in the NAAT-positive patients.^[15]^

The aim of this study is to compare the clinical outcomes of CDI in patients with and without IBD.

## 2. Methods

The study was conducted in Beilinson Hospital at Rabin Medical Center and included adult patients (age of ≥18 years old), admitted between the years 2014 and 2020 in medical or surgical wards with a concurrent diagnosis of IBD. CDI diagnosis was provided using a stool sample and evaluated by the C. DIFF QUIK CHEK COMPLETE assay (TechLab, Blacksburg, VA). This assay detects toxin A, toxin B, and glutamate dehydrogenase antigens. Discrepancy between the toxin and the glutamate dehydrogenase exam was solved using Xpert CD polymerase chain reaction assay (BD Gene-OhmTM Cdiff Assay; Franklin Lakes, NJ). The study was approved by Rabin Medical Center institutional review board (RMC-20-0928). Patients were considered to have *Clostridioides* disease and thus included in the study, were only those with a positive toxin test (defined as a positive ELISA test or positive antigen test together with a positive NAAT for the toxin). Patients with only positive antigen tests and negative toxin results were not included in the study and were not considered to have *Clostridium* disease based on current guidelines and recommendations.^[16,17]^

Clinical data were collected retrospectively using electronic medical charts. Information regarding patients’ general characteristics; background disease; IBD status; laboratory results, clinical presentation, and outcomes were collected electronically. Comorbidities were defined using the modified Charlson comorbidity index.^[18]^ Patients were divided into an IBD group and a non-IBD group depending on IBD diagnosis prior to admission. Follow-up period was 90 days. The primary outcomes were time to resolution of the symptom; 90-day mortality; recurrence of infection; the need for colectomy and intensive care unit admission. Severe CDI was defined as a white blood cell count >15,000 cells/mL or serum creatinine value >1.5 mg/dL. Fulminant CDI was defined as hypotension, shock, or surgery to megacolon.^[19]^ Clinical cure was defined as the first day without diarrhea during the study follow-up. Patients and the public were not involved in any way in the design, conduct, reporting, or dissemination plans of the research. This is due to the retrospective nature of the study. The study was approved by Rabin Medical Center local ethics committee.

### 2.1. Statistical analysis

Statistical analysis was performed using IBM SPSS Statistics 27 (IBM, Armonk, NY). Continuous non-normally distributed variables were presented as median and interquartile range. All patients were matched 1:2 using sex and age as matching variables. Categorical variables and main comparisons of groups and outcomes were compared using chi square test. For adjusted comparison, binary logistic regression was used. All variables found clinically or statically significant were accounted for in the regression. A *P* value <.05 was considered significant.

## 3. Results

Our cohort included 201 patients with CDI, 67 patients with IBD, and 134 patients without IBD. The total study population had a median age of 40.6 and IQR of 29.8 to 68.9, 92/201 were male (45.8%) and 109/201 were female (54.2%), as presented in Table 1. The patients were different in their comorbidities, using the Charlson score, the median of the non-IBD group was 2 [IQR 0;5] versus 0 [0;4] in the IBD group (*P* value <.01). Moreover, the IBD group had more use of steroids in the 3 months prior to admission, 34/67 (59.6%) versus 29/134 (21.6%).

**Table 1:** Patients characteristics.

	Total	IBD group, no. (%)	Control group, no. (%)	*P* value
Patients	201	67	134	
Age in yr, median [IQR]	41 [29; 69]	40.6 [29.8; 68.9]	41 [29; 69]	
Sex				
Male	92 (45.8%)	31 (46.3%)	61 (45.5%)	
Female	109 (54.2%)	36 (53.7%)	73 (54.5%)	
Charlson comorbidity score, median [IQR]	2 [2; 5]	0 [0; 4]	2 [0; 5]	**.002**
Hemoglobin in mg/dL, mean [SD]	11.13 [2.23]	10.6 [2.05]	11.3 [2.28]	.052
CRP, median [IQR]	5.45 [2.1; 13.7]	5.9 [2.8; 13.2]	4.99 [1.8; 13.8]	.486
Creatinine, median [IQR]	0.82 [0.66; 1.18]	0.8 [0.62; 1.08]	0.85 [0.68; 1.35]	.18
Steroid administration	63 (33%)	34 (59.6%)	29 (21.6%)	**<.01**
Fever	54 (26.9%)	34 (50.7%)	20 (14.9%)	**<.01**
Leukocytosis	58 (28.9%)	21 (31.3%)	37 (27.6%)	.582
Acute kidney injury	30 (20.3%)	9 (13.4%)	21 (25.9%)	.06
Hypotension	8 (4.7%)	3 (5.8%)	5 (4.2%)	.701
Recent admission	82 (42.3%)	21 (35%)	61 (45.5%)	.17
Recent antibiotics	73 (39.2%)	38 (71.1%)	35 (26.3%)	**<.01**
Type of CD detection				
ELISA for toxin positive	78 (38.8%)	14 (20.9%)	64 (47.8%)	**<.01**
PCR for Toxin positive	123 (61.2%)	53 (79.1%)	70 (52.2%)	**<.01**
Severe CDI	79 (39.3%)	29 (43.3%)	50 (37.3%)	.414
Fulminant CDI	6 (3%)	3 (4.5%)	3 (2.2%)	.402

CDI = Clostridioides difficile infection, CRP = C-reactive protein, ELISA = enzyme-linked immunosorbent assay, IBD = inflammatory bowel disease, IQR = interquartile range, PCR = polymerase chain reaction, SD = standard deviation.

The IBD group consisted of 33 patients with Crohn disease, 32 patients with ulcerative colitis, and 2 patients with unspecified IBD. Twenty-nine patients (55.8%) had colonic disease, 10 (19.2%) had only ileal involvement and 12 patients (23.1%) had ileocolonic disease. Eleven out of the 67 patients (16.7%) had previous intestinal surgery, 34/67 (59.6%) were on steroids at the time of CDI infection, and 11/67 (19%) used anti-TNF medications. Data is fully presented in Table 2.

**Table 2:** Inflammatory bowel disease characteristics.

	Number (%)
Type of IBD	
Crohn disease	33 (49.3%)
Ulcerative colitis	32 (47.8%)
Not otherwise specified	2 (3%)
Disease involvement	
Colon	29 (55.8%)
Ileum	10 (19.2%)
Ileocolonic disease	12 (23.1%)
Isolated rectal disease	1 (1.9%)
Previous surgery	11 (16.7%)
Recent steroid use	34 (59.6%)
Recent 5ASA use	28 (47.5%)
Recent 6MP use	8 (13.8%)
Recent anti TNF use	11 (19%)
Recent anti integrin use	2 (3.4%)

5ASA = , IBD = inflammatory bowel disease, 6MP = , TNF = .

Clinical presentation and laboratory results were similar between the 2 groups regarding leukocytosis, kidney function, and recent hospitalization. IBD patients were more likely to present with a fever above 38 degrees 50.7% versus 14.9% in the non-IBD group (*P* value <.01). As expected, patients with IBD had more exposure to systemic antibiotics in the 3 months prior to CDI diagnosis, 71.1% versus 26.3% (*P* value <.01). On univariate analysis, patients with IBD were at similar risk to develop severe or complicated CDI 43.3% versus 37.3% (*P* value .414) and 4.5% versus 2.2% (*P* value .402) respectively, as presented in Table 3.

**Table 3:** Treatment and outcomes.

	Total	IBD group, No. (%)	Control group, no. (%)	*P* value
Enteral metronidazole	89 (44.3%)	12 (17.9%)	77 (57.5%)	**<.01**
Vancomycin	121 (60.2%)	44 (65.7%)	77 (57.5%)	.262
Fidaxomicin	15 (7.5%)	9 (13.4%)	6 (4.5%)	**.023**
In-admission mortality	15 (8.1%)	2 (3.8%)	13 (9.8%)	.239
90-d mortality	18 (9%)	6 (9%)	12 (9%)	.987
Intensive care unit admission	10 (5%)	1 (1.5%)	9 (6.7%)	.17
CDI relapse	12 (6.1%)	4 (6.2%)	8 (6%)	1
Length of admission, median [IQR]	6 [2; 15]	4 [2; 9]	7 [2; 21]	.207
Days to symptom resolution, median [IQR]	3.5 [2; 7]	5 [3; 12]	3 [1; 7]	.06

CDI = Clostridioides difficile infection, IBD = inflammatory bowel disease, IQR = interquartile range.

Regarding treatment, 89 of the 201 patients in the cohort were treated with enteral metronidazole – 17.9% in the IBD group versus 57.5% in the non-IBD group. The use of vancomycin was similar between the 2 groups, while fidaxomicin was more commonly utilized in the IBD group with 9 versus 6 patients (*P* value <.05). The median length of hospital stay was 6 days [IQR 2; 15] and the number of days to symptoms resolution was higher in the IBD group with a median of 5 days [IQR 3; 15] versus 3 days [IQR 2; 7], although it failed to reach statistical significance.

**Figure 1.**
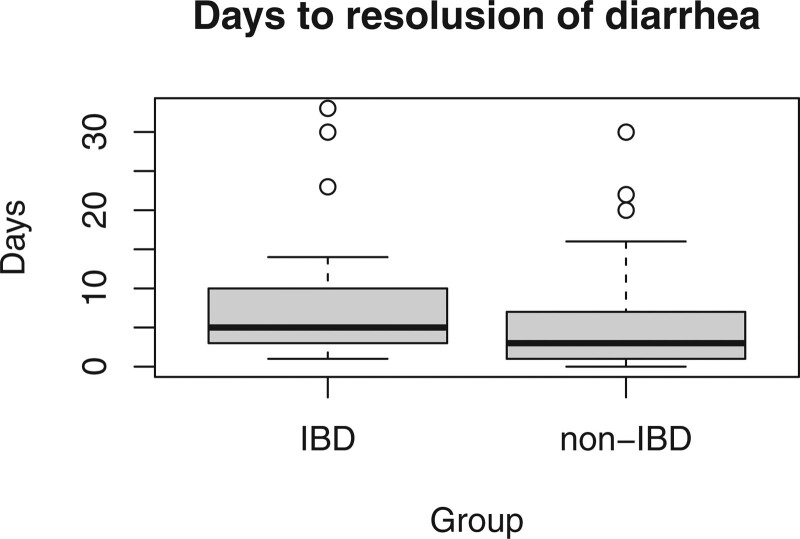
Days to symptom resolution.

Molecular diagnosis was different between the 2 groups with 14/67 (20.9%) being ELISA positive in the IBD group versus 64/134 (47.8%) in the non-IBD group (*P* value <.01). Although the mortality rate was higher in patients with a positive ELISA for toxin test compared to only positive NAAT result, it did not reach statistical significance either in the non-IBD or in the IBD group. No difference was demonstrated between the groups in 90-day mortality, the first event of relapse, hospital mortality, intensive care unit admission, and colectomy rates as presented in Table 3 and Figure 1. In a multivariable analysis, the OR for 90-day mortality was 1.709 (95% CI 0.321–9.905) and the OR for the first event of relapse was 2.89 (95% CI 0.055–1.513), as presented in Table 4.

**Table 4:** Multivariable analysis.

	Odds ratio	95% confidence interval
CDI relapse	0.289	0.055–1.513
90-d mortality	1.709	0.321–9.905
In-hospital mortality	0.198	0.021–1.853

CDI = Clostridioides difficile infection.

## 4. Discussion

Our study compared the clinical characteristics and outcomes of 67 hospitalized CDI patients with IBD to those without IBD treated in the same center. The study population in the 2 groups was different in their basic characteristics. Patients with IBD had fewer comorbidities but clinically presented with more cases of fever; had more use of antibiotics prior to admission and had higher chances of being treated with steroids before admission.

The most common diagnostic modality in IBD patients was ELISA negative and NAAT for toxin positive. Although recent evidence shows a higher mortality rate with ELISA-positive patients compared with polymerase chain reaction,^[15]^ our cohort did not demonstrate any difference between the groups. Although many studies describe the association of ELISA-positive patients with more severe diseases, this finding might be related to disease burden and other unmeasured disease-related features that can be altered in a colon with IBD pathology. Nonetheless, NAAT-positive patients are considered to have CDI based on current guidelines.^[16,17]^ Previous studies demonstrated that patients with IBD have a negative clinical outcome upon acquiring *Clostridioides* infection.^[20–22]^ Furthermore, the best medical approach for a patient with IBD and CDI is an issue of frequent clinical dilemma. The decision to treat with immunosuppression alongside antibiotics is a matter of ongoing debate. In a survey of 169 gastroenterologists from the USA and Canada, a significant disagreement was described in the common practice for these populations of patients.^[23]^

The clinical questions that arise from the current evidence are whether CDI serves as a prognostic factor accounting for the severity of IBD or whether the patients’ outcomes are derived from the infection itself. The acquisition of *Clostridioides* infection in the IBD population is considered a risk factor for negative clinical outcomes.^[21]^ This study aimed to evaluate the patient’s outcomes from the CDI perspective, comparing the IBD patients to patients without IBD. Interestingly, when controlling for age and sex, we found similar rates of mortality and the risk of relapse between the IBD and non-IBD patients. While the study groups were different in their nature and risk factors, with respect to CDI, they had similar outcomes. This observation further establishes that CDI in the IBD population represents similar risk for adverse outcomes as the non-IBD population and needs to be treated and addressed accordingly.

Our study has several limitations. The cohort presented in the study was composed of a small and heterogenic population of patients that had different medical treatments for the *Clostridioides* infection. Patients with IBD were treated more frequently with vancomycin and fidaxomicin compared with non-IBD patients. In spite of that, all patients were treated according to published guidelines^[19]^ and the treatment regimen escalated with regard to disease severity. Furthermore, fidaxomicin is a novel anti-CDI antibiotic that was assessed for IBD patients in a clinical trial only for pharmacokinetics.^[24]^ The prospective treatment of fidaxomicin in IBD patients might have a significant potential benefit due to its microbiome-friendly properties and needs to be further assessed in the IBD population. Lastly, in this study, we examined the antibiotic treatment for CDI in IBD patients but did not explore the treatment regimens for IBD before and during hospitalization and so the effect of IBD treatment on the course of CDI and vice versa was not addressed.

In conclusion, patients with IBD that present with diarrhea and concomitant CDI have similar rates of mortality and relapse compared with patients without IBD. Treating physicians should be alert to the possibility of CDI and treat it accordingly.

## Author contributions

**Conceptualization:** Genady Drozdinsky, Jihad Bishara, Noa Eliakim-Raz.

**Data curation:** Genady Drozdinsky, Alaa Atamna, Haim Ben-Zvi.

**Formal analysis:** Genady Drozdinsky.

**Investigation:** Genady Drozdinsky, Noa Eliakim-Raz.

**Methodology:** Genady Drozdinsky, Noa Eliakim-Raz.

**Supervision:** Jihad Bishara, Noa Eliakim-Raz.

**Writing – original draft:** Genady Drozdinsky.

**Writing – review & editing:** Hagar Banai, Jihad Bishara, Noa Eliakim-Raz.

## References

[1] Burke KE Lamont JT. Clostridium difficile infection: a worldwide disease. Gut Liver. 2014;8:1–6. 24516694 10.5009/gnl.2014.8.1.1 PMC3916678

[2] Antibiotic Resistance Threats in the United States, 2019. Centers for Disease Control and Prevention (U.S.). 2019.

[3] Tavakoli P Vollmer-Conna U Hadzi-Pavlovic D. A review of inflammatory bowel disease: a model of microbial, immune and neuropsychological integration. Public Health Rev. 2021;42:1603990. 34692176 10.3389/phrs.2021.1603990 PMC8386758

[4] Molodecky NA Soon IS Rabi DM. Increasing incidence and prevalence of the inflammatory bowel diseases with time, based on systematic review. Gastroenterology. 2012;142:46–54.e42; quiz e30. 22001864 10.1053/j.gastro.2011.10.001

[5] Sehgal K Yadav D Khanna S. The interplay of *Clostridioides difficile* infection and inflammatory bowel disease. Therap Adv Gastroenterol. 2021;14:17562848211020217562848211020284. 10.1177/17562848211020285 PMC8170344 34104215

[6] Rodemann JF Dubberke ER Reske KA. Incidence of Clostridium difficile infection in inflammatory bowel disease. Clin Gastroenterol Hepatol. 2007;5:339–44. 17368233 10.1016/j.cgh.2006.12.027

[7] Capurso L Koch M. Infezione da <em>Clostridium difficile</em> e malattie infiammatorie croniche intestinali. Recenti Prog Med. 2021;112:42–55. 33576350 10.1701/3551.35256

[8] Viazis N Pontas C Karmiris K. Prevalence of Clostridium difficile infection among hospitalized inflammatory bowel disease patients in Greece. Eur J Gastroenterol Hepatol. 2019;31:773–6. 30973416 10.1097/MEG.0000000000001414

[9] Khanna S. Management of *Clostridioides difficile* infection in patients with inflammatory bowel disease. Intest Res. 2021;19:265–74. 32806873 10.5217/ir.2020.00045 PMC8322030

[10] Jodorkovsky D Young Y Abreu MT. Clinical outcomes of patients with ulcerative colitis and co-existing clostridium difficile infection. Dig Dis Sci. 2010;55:415–20. 19255850 10.1007/s10620-009-0749-9

[11] Chen XL Deng J Chen X. High incidence and morbidity of Clostridium difficile infection among hospitalized patients with inflammatory bowel disease: a prospective observational cohort study. J Dig Dis. 2019;20:460–6. 31278840 10.1111/1751-2980.12798

[12] Beniwal-Patel P Stein DJ Munoz-Price LS. The juncture between *Clostridioides difficile* infection and inflammatory bowel diseases. Clin Infect Dis. 2019;69:366–72. 30689770 10.1093/cid/ciz061

[13] Gupta A Wash C Wu Y. Diagnostic modality of *Clostridioides difficile* infection predicts treatment response and outcomes in inflammatory bowel disease. Dig Dis Sci. 2020. 10.1007/s10620-020-06205-6 32207033

[14] Wilcox MH Rahav G Dubberke ER. Influence of diagnostic method on outcomes in phase 3 clinical trials of bezlotoxumab for the prevention of recurrent *Clostridioides difficile* infection: a post hoc analysis of MODIFY I/II. Open Forum Infect Dis. 2019;6:ofz293. 31375837 10.1093/ofid/ofz293 PMC6677672

[15] Avni T Babich T Ben-Zvi H. Molecular-based diagnosis of Clostridium difficile infection is associated with reduced mortality. Eur J Clin Microbiol Infect Dis. 2018;37:1137–42. 29627950 10.1007/s10096-018-3228-4

[16] Johnson S Lavergne V Skinner AM. Clinical practice guideline by the Infectious Diseases Society of America (IDSA) and Society for Healthcare Epidemiology of America (SHEA): 2021 focused update guidelines on management of *Clostridioides difficile* infection in adults. 10.1093/cid/ciab549 34164674

[17] van Prehn J Reigadas E Vogelzang EH. European society of clinical microbiology and infectious diseases: 2021 update on the treatment guidance document for *Clostridioides difficile* infection in adults. Clin Microbiol Infect. 2021;27:S1–S21. 10.1016/j.cmi.2021.09.038 34678515

[18] Etienne A Esterni B Charbonnier A. Comorbidity is an independent predictor of complete remission in elderly patients receiving induction chemotherapy for acute myeloid leukemia. Cancer. 2007;109:1376–83. 17326052 10.1002/cncr.22537

[19] McDonald LC Gerding DN Johnson S. Clinical practice guidelines for Clostridium difficile infection in adults and children: 2017 update by the Infectious Diseases Society of America (IDSA) and Society for Healthcare Epidemiology of America (SHEA). Clin Infect Dis. 2018;66:987e1–994. 29562266 10.1093/cid/ciy149

[20] Issa M Vijayapal A Graham MB. Impact of Clostridium difficile on inflammatory bowel disease. Clin Gastroenterol Hepatol. 2007;5:345–51. 17368234 10.1016/j.cgh.2006.12.028

[21] Tariq R Law CCY Khanna S. The impact of Clostridium difficile infection on mortality in patients with inflammatory bowel disease: a systematic review and meta-analysis. J Clin Gastroenterol. 2019;53:127–33. 29206751 10.1097/MCG.0000000000000968

[22] Management of Clostridium difficile infection in inflammatory bowel disease: Expert review from the Clinical Practice Updates Committee of the AGA Institute - ClinicalKey. 10.1016/j.cgh.2016.10.024 28093134

[23] Practice of gastroenterologists in treating flaring inflammatory bowel disease patients with Clostridium difficile: antibiotics alone or combined antibiotics/immunomodulators? - PubMed. 10.1002/ibd.21514 21674710

[24] Högenauer C Mahida Y Stallmach A. Pharmacokinetics and safety of fidaxomicin in patients with inflammatory bowel disease and *Clostridium difficile* infection: an open-label Phase IIIb/IV study (PROFILE). J Antimicrob Chemother. 2018;73:3430–41. 30260412 10.1093/jac/dky368

